# Blue light reflectance imaging in non-perfusion areas detection: insights from multimodal analysis

**DOI:** 10.1186/s40942-024-00602-z

**Published:** 2024-11-04

**Authors:** Ricardo Leitão Guerra, Gabriel Castilho Sandoval Barbosa, Cezar Leitão Guerra, Emmerson Badaro, Luiz Roisman, Luiz Filipe Lucatto, Eduardo Novais

**Affiliations:** 1 Orbit Ophthalmo Learning, Salvador, Brazil; 2 Department of Ophthalmology, University of São Paulo, Tamandaré Street, 655, Apartment 106, São Paulo, 01525-001 Brazil; 3 Leitão Guerra - Oftalmologia, Salvador, Brazil

## Abstract

**Design:**

A retrospective, cross-sectional image analysis using a convenience sample.

**Subjects:**

Five cases selected based on the availability of comprehensive imaging data.

**Methods:**

This study involved a retrospective review of images from five cases, focusing on the use of retinal monochromatic blue light reflectance (BLR) imaging to detect non-perfusion areas. Two cases of sickle-cell retinopathy demonstrated peripheral retinal non-perfusion identified through widefield fluorescein angiography. Three other cases—one with branch retinal vein occlusion, one with branch retinal artery occlusion, and one presenting paracentral acute middle maculopathy showed focal macular non-perfusion detected by structural OCT and OCTA. The areas of nonperfused retinal tissue, confirmed by fluorescein angiography, OCT, and OCTA, were then correlated with findings from the BLR image. This correlation aimed to identify any potential associations between these imaging modalities.

**Main outcome measures:**

Enhance understanding of the utilization of retinal monochromatic BLR images as a non-perfusion biomarker.

**Results:**

The perfusion defects identified through fluorescein angiography were qualitatively correlated with hypo-reflective regions observed in the BLR images. A notable correlation was also observed between the OCTA deep capillary plexus findings and the BLR images. Additionally, areas of retinal thinning identified on structural OCT thickness maps corresponded with the hypo-reflective regions in the BLR images. This indicates the potential of BLR in identifying non-perfused retinal areas.

**Conclusions:**

This study reinforces the evidence, through OCT, OCTA, and angiographic correlation, that the BLR can effectively identify areas of retinal non-perfusion in a non-invasive manner. Further research is warranted to assess the method’s sensitivity, specificity, and limitations. While the interaction of blue light with the retina, leading to specular reflections and scattering, is established, this research represents a pioneering effort in suggesting which specific retinal structures may be implicated in this phenomenon. This novel insight opens avenues for deeper exploration into the underlying mechanisms and potential clinical applications of utilizing the BLR imaging technique for assessing retinal vascular abnormalities.

## Introduction

Retinal imaging has undergone significant advancements over the past few decades, enabling clinicians and researchers to visualize the ocular fundus with remarkable detail and precision. Among the various imaging modalities, monochromatic fundus photography, particularly blue light reflectance (BLR), has emerged as a promising technique for enhancing the visualization of specific retinal layers and diseases [1].

Following the same principle as filters, color channels are generated by decomposing the color image into separate monochromatic images that represents the interaction of light of a specific wavelength spectrum with retinal tissue [1, 2]. The longer the wavelength, the deeper the penetration into the tissue, and for this reason, the BLR is primarily used for studying the inner structures of the neurosensory retina. It occurs because the blue light spectrum is absorbed by the melanin in the retinal pigment epithelium (RPE) and choroid, the macular pigment such as lutein and zeaxanthin, and the blood, resulting in a dark image of these structures, while it is reflected by the retinal tissue, specially the inner layers [1–4].

Previous studies, including preliminary findings from our research group, have suggested the potential of retinal BLR imaging to identify a novel biomarker related to non-perfusion areas within the retina [5–8]. Our prior publication detailed a single case where BLR imaging revealed patterns consistent with non-perfused regions, although without angiographic confirmation to substantiate these observations [8].

The clinical significance of non-perfusion in retinal diseases cannot be overstated. Non-perfusion areas are indicative of ischemia, which can lead to severe visual impairment and are characteristic of various retinal vascular disorders, including diabetic retinopathy, retinal vein occlusion, and retinal artery occlusion. Identifying these areas accurately and non-invasively is crucial for early diagnosis, monitoring disease progression, and tailoring treatment strategies to prevent irreversible damage.

Biomarkers play a pivotal role in the clinical management of retinal diseases, offering measurable indicators of disease state, progression, and response to therapy [9]. In ophthalmology, the quest for non-invasive biomarkers is particularly pertinent due to the delicate nature of ocular tissues. Non-invasive imaging biomarkers that can accurately detect and quantify retinal non-perfusion would represent a significant leap forward, reducing reliance on invasive procedures like fluorescein angiography (FA), which, despite its diagnostic utility, carries risks of allergic reactions and nephrotoxicity [10, 11].

Our current study builds upon our initial observations by exploring the application of BLR imaging in identifying non-perfusion biomarkers more comprehensively. Through the integration of multimodal retinal imaging, including wide-field FA, optical coherence tomography (OCT) and OCT angiography (OCTA), we aim to explore the specific characteristics of BLR imaging in detecting retinal non-perfusion across various retinal pathologies. This research not only seeks to validate previous observations but also to investigate the underlying mechanisms that contribute to BLR patterns in the context of retinal ischemia.

## Methods

This study was designed as a retrospective, cross-sectional imaging analysis using a convenience sample. The study reviewed images from five cases selected based on the availability of comprehensive imaging data, including high-quality color fundus (CF) images, wide-field FA, OCT, OCTA, and red, green, and blue (RGB) reflectance (generated from the respective CF images monochromatic channels). While not all cases had every exam available, we utilized all the imaging data at our disposal for each case to ensure the most comprehensive analysis possible. Cases were chosen to represent a spectrum of retinal non-perfusion scenarios to explore the utility of BLR imaging against established diagnostic modalities.

CF and FA images were acquired using Clarus 700 fundus camera and OCT and OCTA were captured using Cirrus 6000, both from Carl Zeiss Meditec, Dublin, California, USA. Forum-Viewer software version 4.4 for MacOS (Carl Zeiss Meditec, Dublin, California, USA) was used to split RGB channels, analyze OCT and OCTA scans, and correlate tomographic and fundus images. This software allows the operator to overlay OCT and OCTA scans (both B-scans and en-face images) over the accurate area of a CF, RGB channels, or FA image. By doing this, the operator can correlate abnormal areas on both exams.

Two independent ophthalmologists (RLLG and CLLG) graded the images. In cases of disagreement, a third reviewer adjudicated the findings to reach a consensus. The analysis focused on comparing the perfusion defects identified by FA or OCT-A with the hypo-reflective areas visible in the BLR images. The study specifically examined the qualitative correlation between these hypo-reflective areas and the underlying retinal pathology, as indicated by structural changes on OCT and the capillary plexus visibility on OCTA. Also, the green light reflectance (GLR) and the red light reflectance (RLR) images were accessed for abnormalities, and structural changes on the OCT were analyzed.

## Results

Our retrospective analysis encompassed imaging data from five distinct and illustrative cases: Two cases of sickle-cell retinopathy, one case of branch retinal vein occlusion (BRVO), one case of branch retinal artery occlusion (BRAO), and one case presenting the consequences of a previous paracentral acute middle maculopathy (PAMM) in a patient with no history of previous retina disease. There was complete concordance between the two independent graders in all cases evaluated. The findings from each case are presented below, alongside the corresponding figure legends, to illustrate the diagnostic concordance between traditional and novel imaging techniques.

### Case 1 and 2: peripheral non-perfusion detected by Fluorescein Angiography

**Fig. 1:**
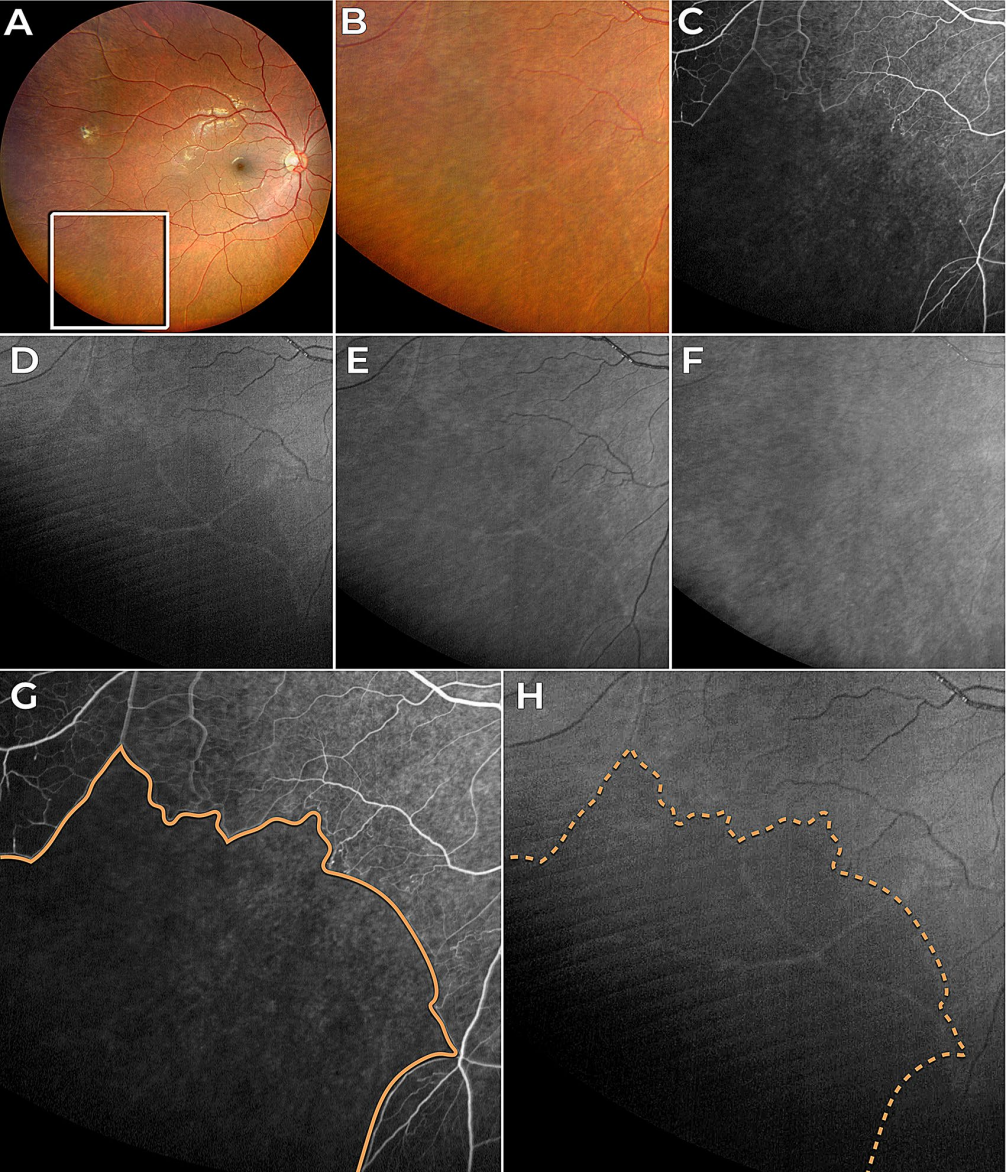
Case 1 - Sickle-cell retinopathy. (**A**) Color fundus image highlighting the location of the nonperfused area in the retina (white square). (**B** Close-up view of the color fundus image showing the nonperfused area. (**C**) Fluorescein angiography confirming the nonperfused area. (**D**) Blue light reflectance image showing hyporeflectance of the nonperfused area, with a minor discrepancy observed in the upper left corner, where a small area does not exhibit hyporeflectance. (**E**) Green light reflectance image showing a subtle difference between the non-perfusion and the healthy area. (**F**) Red light reflectance image. (**G**) Fluorescein angiography with continuous line delimiting non-perfusion region. (**H**) Blue light reflectance image with dashed line delimiting non-perfusion region

**Fig. 2:**
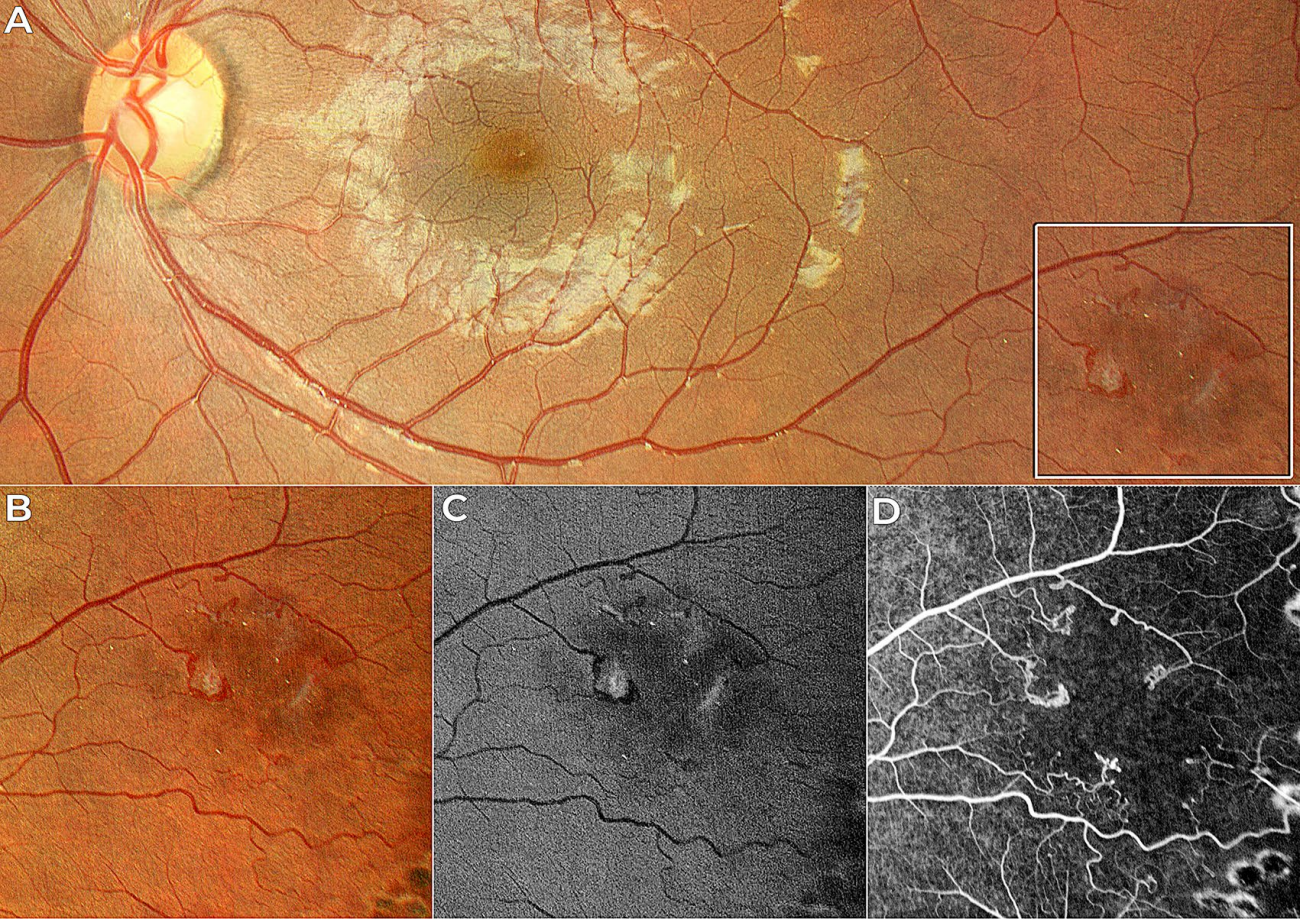
Case 2 - Sickle-cell retinopathy. (**A**) Color fundus image highlighting the location of the nonperfused area in the retina (white square). (**B**) Close-up view of the color fundus image showing the nonperfused area. (**C**) Blue light reflectance image showing hyporeflectance of the nonperfused area. (**D**) Fluorescein angiography confirming the nonperfused area

In the first two cases (Figs. 1 and 2), peripheral retinal non-perfusion due to sickle-cell retinopathy was identified using wide-field FA. The perfusion defects observed in the FA images were qualitatively correlated with hyporeflectant areas in the BLR images. The BLR allows the visualization of capillary non-perfused areas, which appear as dark patches contrasting with the grayish look of the perfused retina. In Fig. 1 shows a small non-perfused area on FA that does not match the BLR image, which will be discussed herein.

### Case 3: focal macular non-perfusion detected by OCTA

**Fig. 3:**
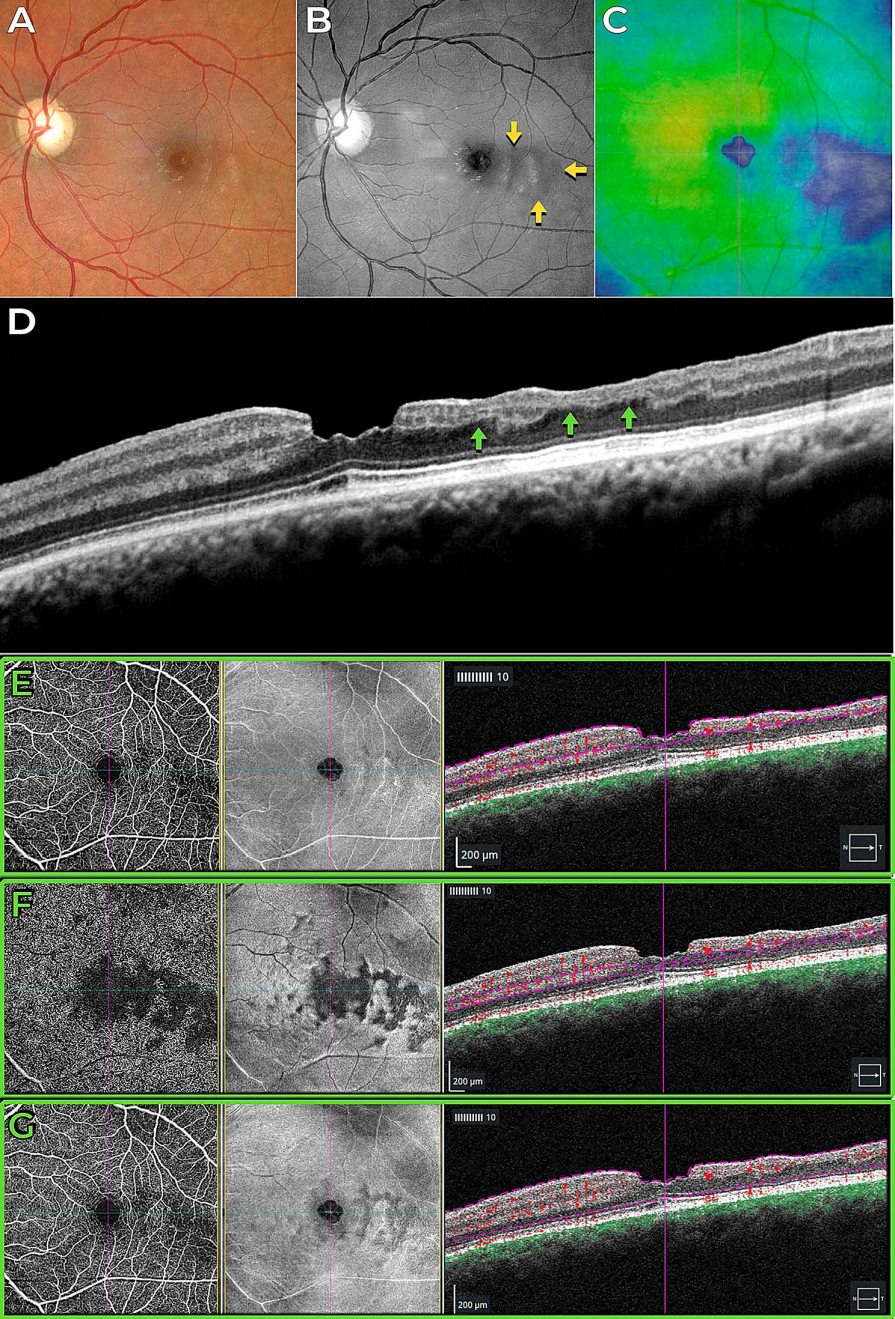
Case 3 - Previous paracentral acute middle maculopathy in a patient with no history of previous retinal disease. (**A**) Color fundus image. (**B**) Blue light reflectance image displaying hyporeflectance of the nonperfused area (indicated by yellow arrows). (**C**) Structural OCT thickness map indicating retinal thinning, represented by cold colors. (**D**) B-scan OCT revealing inner retinal thinning (highlighted by green arrows). OCT angiography and structural en-face maps, along with segmentation overlays on the B-scan OCT images, provide detailed insights into retinal pathology. (**E**) The superficial capillary plexus reveals discrete abnormalities localized to the affected area. (**F**) In contrast, the deep capillary plexus exhibits a pronounced reduced flow signal corresponding to the affected area. (**G**) Analysis of the full retina segmentation reveals mild abnormalities within the affected area, contributing to a comprehensive understanding of the retinal vasculature and structural changes associated with the pathology

**Fig. 4:**
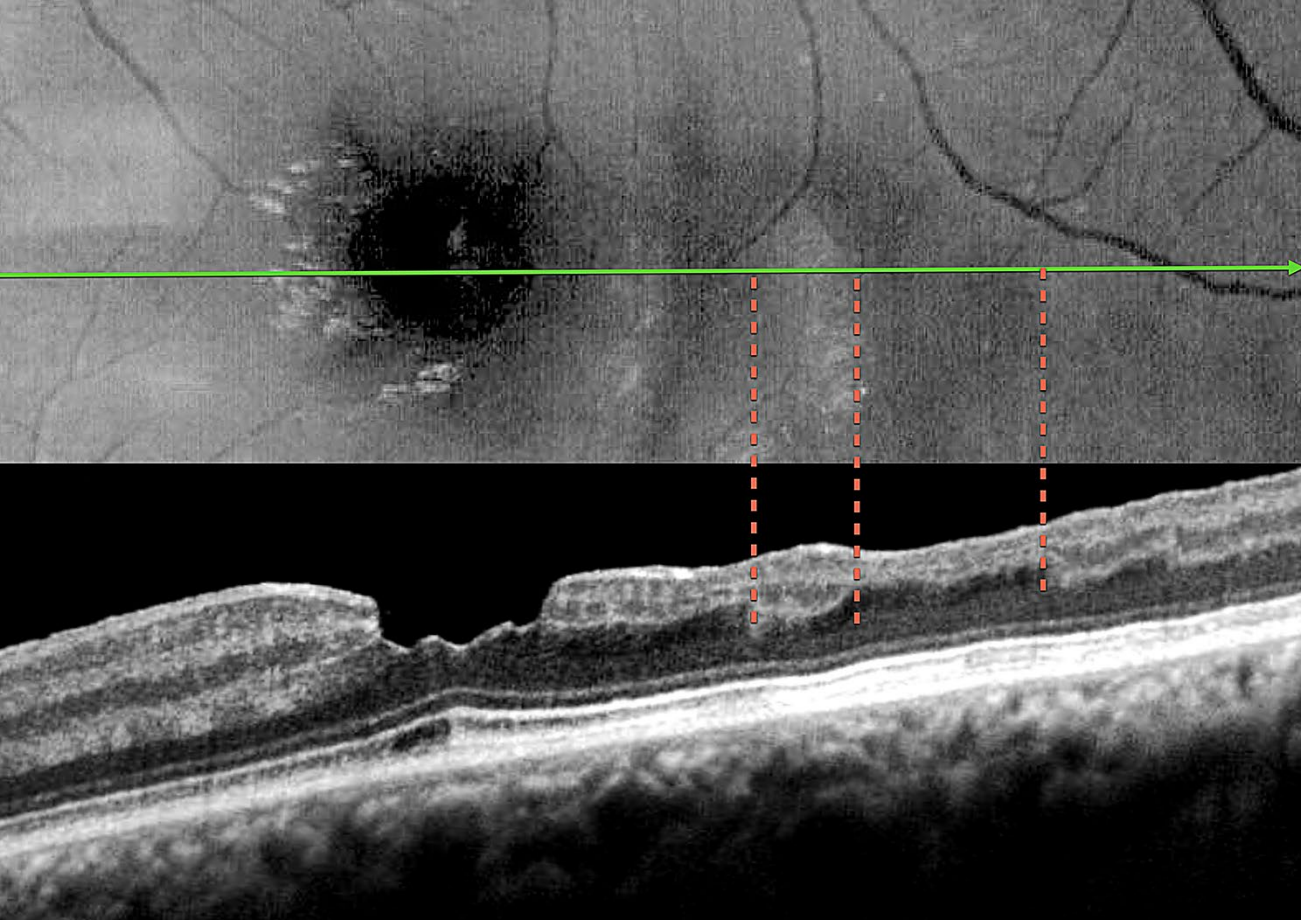
Case 3 - Correlation of the blue light hyporeflectance areas to the OCT B-Scan inner retinal abnormalities. Notice the absence of inner nuclear and outer plexiform layers in the areas of hyporeflectance

The third case presented a focal macular non-perfusion area as a consequence of PAMM in a patient with no history of previous retina disease, which was detected by OCTA (Figs. 3 and 4). The OCTA findings of deep capillary plexus (DCP) non-perfusion, despite normal superficial capillary plexus (SCP) perfusion, were sharply correlated with hyporeflectant areas in the BLR images. Additionally, structural OCT thickness maps revealed retinal thinning in regions corresponding to the hyporeflectant areas observed in the BLR images. In addition, the hyporeflectant areas observed in the BLR images correlate with the absence of an identifiable inner nuclear layer (INL) and outer plexiform layer (OPL) in OCT scans.

### Case 4: branch retinal vein occlusion

**Fig. 5:**
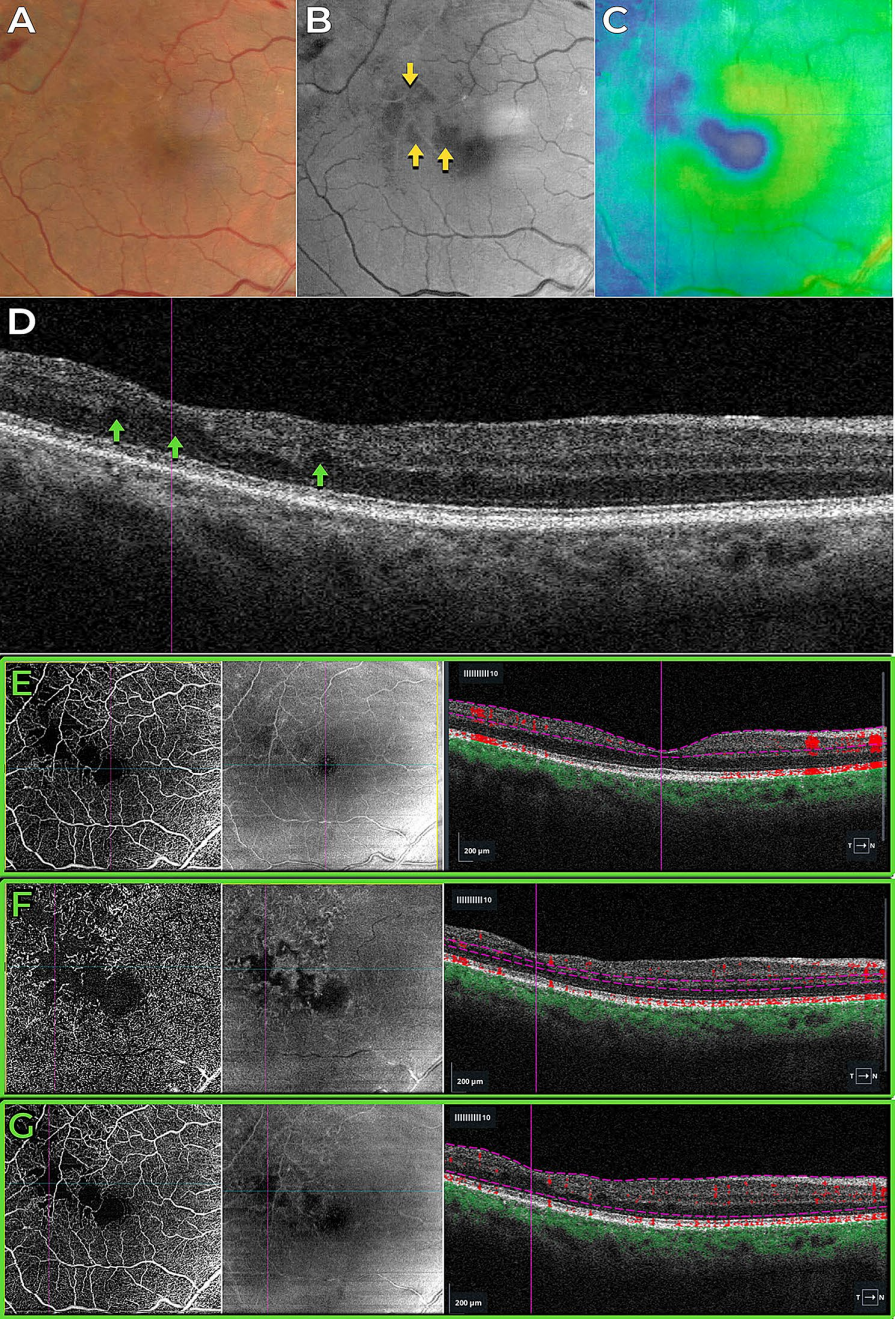
Case 4 - Branch Retinal Vein Occlusion. (**A**) Color fundus image. (**B**) Blue light reflectance image displaying hyporeflectance of the nonperfused area (indicated by yellow arrows). (**C**) Structural OCT thickness map indicating retinal thinning, represented by cold colors. (**D**) B-scan OCT revealing inner retinal thinning (highlighted by green arrows). OCT angiography and structural en-face maps, along with segmentation overlays on the B-scan OCT images, provide detailed insights into retinal pathology. (**E**) The superficial capillary plexus reveals discrete abnormalities localized to the affected area. (**F**) In contrast, the deep capillary plexus exhibits a pronounced reduced flow signal corresponding to the affected area. (**G**) Analysis of the full retina segmentation reveals mild abnormalities within the affected area, contributing to a comprehensive understanding of the retinal vasculature and structural changes associated with the pathology

**Fig. 6:**
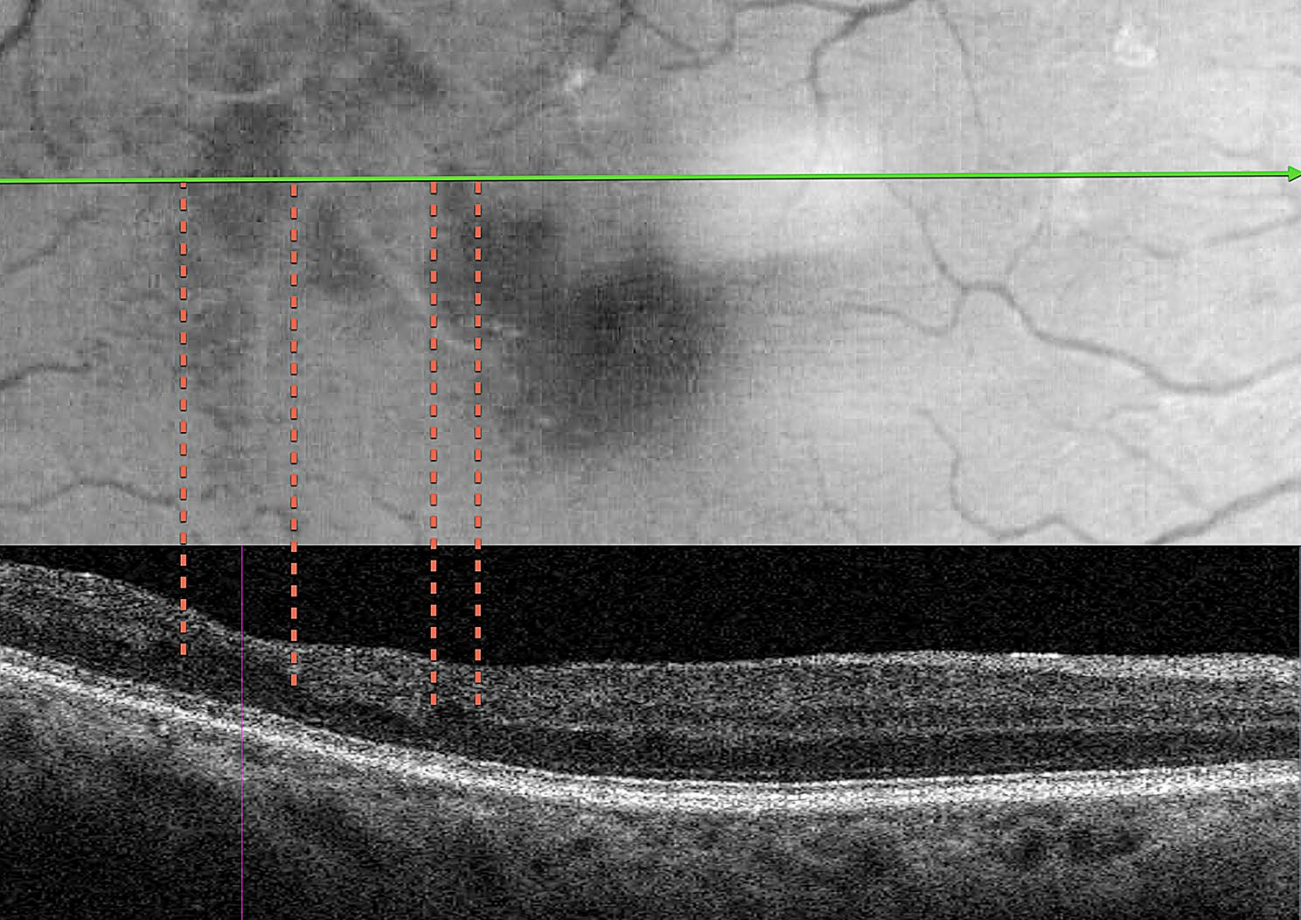
Case 4 - Correlation of the blue light hyporeflectance areas to the OCT B-Scan inner retinal abnormalities. Notice the absence of inner nuclear and outer plexiform layers in the areas of hyporeflectance

The fourth case also presented a focal macular non-perfusion area detected by OCTA, where both the superficial and deep capillary plexus showed a pronounced flow signal loss corresponding to the affected area of the BRVO (Figs. 5 and 6). The OCTA findings correlated well with the hyporeflectant areas observed in the BLR images, as shown in Fig. 5B. In addition, the structural OCT thickness maps revealed retinal thinning in the regions corresponding to these hyporeflectant areas in the BLR images. The correlation of the BLR image with the structural OCT B-Scan evinces that the hyporeflectant areas of the BLR overlap the absence of INL and OPL areas in the OCT.

### Case 5: branch retinal artery occlusion

**Fig. 7:**
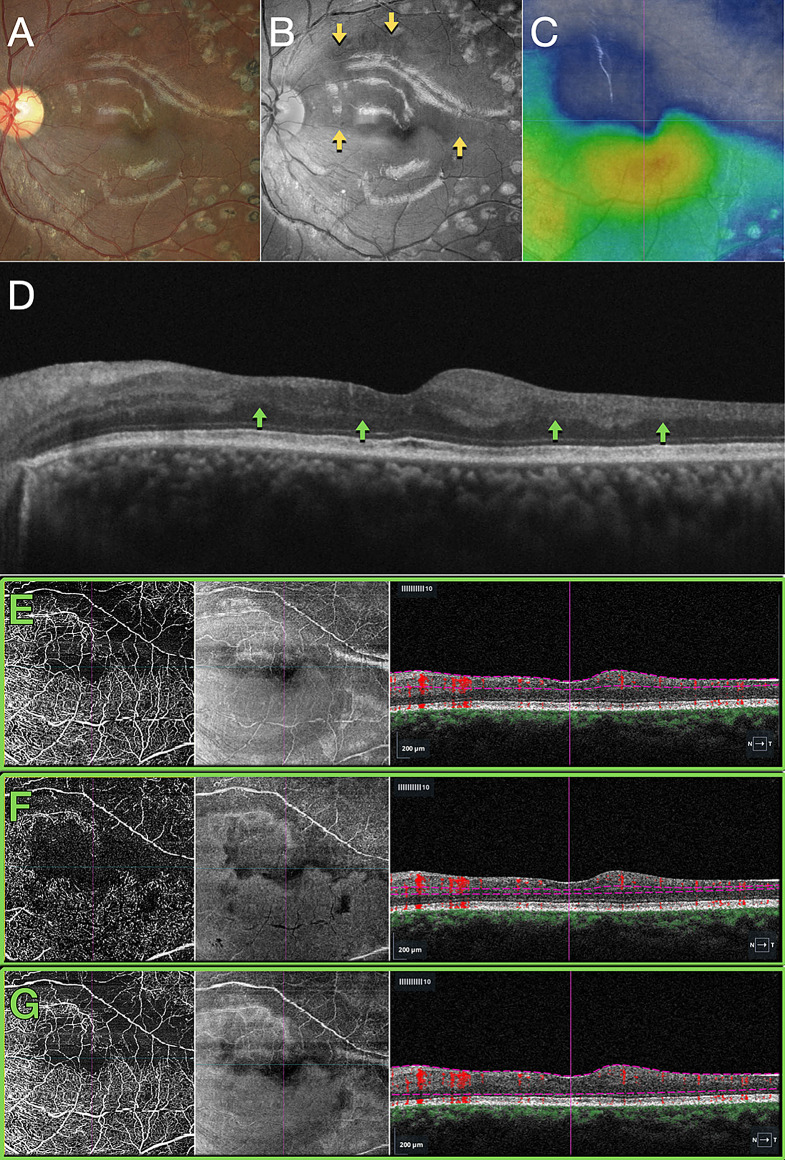
Case 5 - Branch Artery Vein Occlusion. (**A**) Color fundus image. (**B**) Blue light reflectance image displaying hyporeflectance of the nonperfused area (indicated by yellow arrows). (**C**) Structural OCT thickness map indicating retinal thinning, represented by cold colors. (**D**) B-scan OCT revealing inner retinal thinning (highlighted by green arrows). OCT angiography and structural en-face maps, along with segmentation overlays on the B-scan OCT images, provide detailed insights into retinal pathology. (**E**) The superficial capillary plexus reveals discrete abnormalities localized to the affected area. (**F**) In contrast, the deep capillary plexus exhibits a pronounced reduced flow signal corresponding to the affected area. (**G**) Analysis of the full retina segmentation reveals mild abnormalities within the affected area, contributing to a comprehensive understanding of the retinal vasculature and structural changes associated with the pathology

**Fig. 8:**
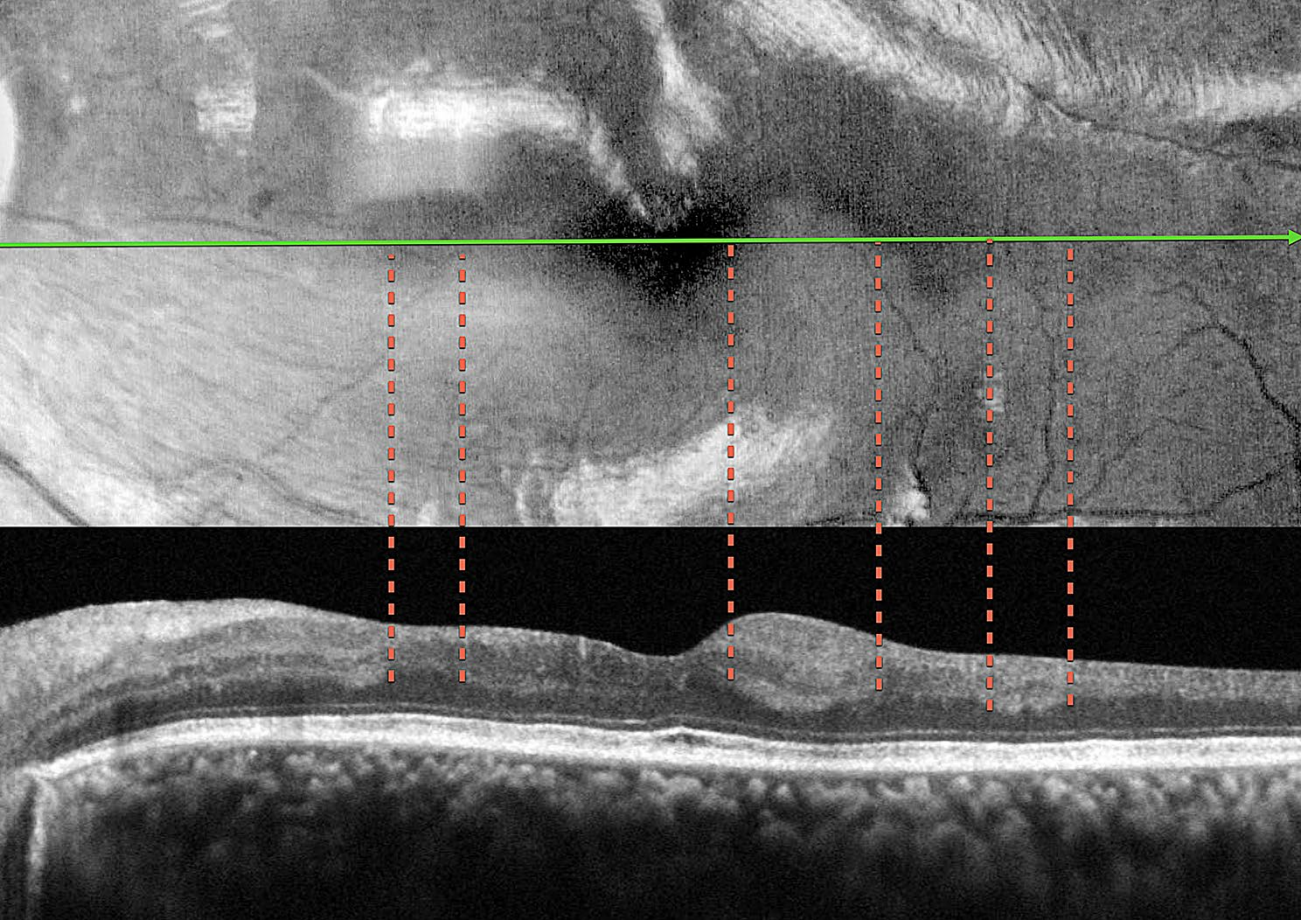
Case 5 - Correlation of the blue light hyporeflectance areas to the OCT B-Scan inner retinal abnormalities. Notice the absence of inner nuclear and outer plexiform layers in the areas of hyporeflectance

In the fifth case, a focal macular non-perfusion area was detected by OCTA, mirroring the findings of the fourth case (Figs. 7 and 8). A pronounced flow signal loss defect was observed in both the superficial and deep capillary plexus, corresponding to the affected region of the BRAO. These OCTA results correlated with the hypo-reflective areas seen in the BLR images. Additionally, structural OCT thickness maps revealed retinal thinning in areas that matched the hyporeflectant regions in the BLR images. Moreover, the hyporeflectant areas in the BLR images were associated with the absence of a distinguishable INL and OPL in the OCT scans. Table 1 summarizes the five cases, highlighting key findings based on different imaging techniques.

**Table 1:** The table below summarizes the five cases presented in the manuscript, highlighting key findings based on different imaging techniques

Case No.	Diagnosis	Imaging Techniques	Non-Perfusion Location	Findings on BLR	Findings on FA/OCTA/Structural OCT
Case 1	Sickle-Cell Retinopathy	FA, Blue, Green, and Red Light Reflectances	Peripheral Retina	Hyporeflectant areas matching non-perfused areas in the FA	FA identified peripheral non-perfusion; BLR hyporeflectance correlated with non-perfused regions
Case 2	Sickle-Cell Retinopathy	FA, Blue, Green, and Red Light Reflectances	Peripheral Retina (Focal)	Hyporeflectanct areas matching non-perfused areas in the FA	FA identified peripheral focal non-perfusion; BLR hyporeflectance correlated with non-perfused regions
Case 3	Previous Paracentral Acute Middle Maculopathy (PAMM) in a Heathy Patient	OCTA, BLR, Structural OCT	Macula (Focal)	Hyporeflectant areas correlate with non-perfusion and retinal thinning in the DCP, and with the absence of INL and OPL areas	OCTA revealed DCP non-perfusion; Structural OCT showed thinning, INL, and OPL absence in affected areas in the BLR hyporeflectant areas
Case 4	Branch Retinal Vein Occlusion (BRVO)	OCTA, BLR, Structural OCT	Macula (Focal)	Hyporeflectant areas correlate with non-perfusion and retinal thinning in the DCP, and with the absence of INL and OPL areas	OCTA revealed non-perfusion in both SCP and DCP; Structural OCT showed retinal thinning and INL/OPL absence in the BLR hyporeflectant areas
Case 5	Branch Retinal Artery Occlusion (BRAO)	OCTA, BLR, Structural OCT	Macula (Focal)	Hyporeflectant areas correlate with non-perfusion and retinal thinning, and with the absence of INL and OPL areas	OCTA revealed non-perfusion in both SCP and DCP; Structural OCT showed thinning, with INL, and OPL absence in the BLR hyporeflectant areas

FA: Fluorescein Angiography; OCT: Optical Coherence Tomography; OCTA: OCT-Angiography; BLR: Blue Light Reflectance; Deep Capillary Plexus: DCP; Superficial Capillary Plexus: SCP; Inner Nuclear Layer: INL; Outer Plexiform Layer: OPL

## Discussion

Our analysis of five cases demonstrated a correspondence between perfusion defects, identified by FA and OCTA, and hyporeflectant areas in BLR images. Also, the study was able to identify the absence of the INL and OPL at these hyporeflectant areas in all five cases with available OCT scans.

This discussion will elaborate on the anatomical and physiological underpinnings that might support this observation, integrating knowledge from the available literature and the inherent properties of retinal layers as they interact with blue light. The findings emphasize the utility of non-invasive imaging techniques in assessing retinal vascular health, supported by the anatomical and physiological characteristics of retinal layers and their interaction with blue light.

Monochromatic fundus imaging using different wavelengths of light interacts with the retina in various ways, highlighting specific structures and revealing details not visible in CF images. Understanding these interactions is crucial for optimizing diagnostic imaging in ophthalmology [1].

The GLR provides excellent overall retinal contrast. It is partially reflected by retinal pigmentation and absorbed by blood, making it particularly effective for viewing retinal blood vessels, hemorrhages, drusen, and exudates [12]. In contrast, the RLR is instrumental in visualizing deeper structures within the eye. Red light wavelength penetrates deeper into the retina and choroid, revealing more of the choroidal pattern. This is useful for visualizing pigmentary disturbances, choroidal ruptures, choroidal nevi, and choroidal melanomas [12].

Blue light wavelength enhances the visibility of the anterior retinal layers, which are typically nearly transparent under white light. This wavelength is highly effective in highlighting semitransparent structures due to its high scattering properties [1]. The blue light is strongly absorbed by macular pigment such as lutein and zeaxanthin, hemoglobin within the blood vessels, and melanin at the level of the RPE and choroid, the latter creating a dark background that accentuates specular reflections and scattering in the anterior retinal layers [1].

Historically, this method is associated with increasing visibility and aids in the identification of the retinal nerve fiber layer, internal limiting membrane, retinal folds, cysts, and epiretinal membranes [1]. However, recent evidence shows that the benefits of BLR are not limited to these conditions, playing an important role in the evaluation of other retinal conditions such as Type 2 Macular Telangiectasia [13], subretinal drusenoid deposits [14], and central serous chorioretinopathy [15].

Previous research, including our preliminary study, has shown the blue channel’s ability to identify retinal arteriolosclerosis, sclerosed venules, and areas of retinal ischemia [5–8]. Hyporeflectant areas on BLR obtained through confocal scanning laser ophthalmoscopy (cSLO) showed a high correlation when compared to FA in patients with diabetic retinopathy (DR) [5, 7] and retinal vein occlusion (RVO) [5]. Also, an association between retinal thinning and disorganization in OCT was found [7]. This research also noticed that the area of nonperfusion appeared larger on FA than BLR [7].

In attempting to explain the possible cause of hyporeflectant areas in the non-perfused retina, it was suggested that this phenomenon may occur due to reduced blood flow, as the authors proposed that blue light is reflected by red blood cells (RBC), and the absence of RBC would result in a hyporeflectant appearance [5]. However, blue light is not reflected by RBC, but rather absorbed by the hemoglobin present in RBC, which accounts for the hyporeflectance of blood vessels and hemorrhages in the images captured by this method [1].

Another recently raised hypothesis was the reduction in thickness and disorganization of the retina, observed in OCT, as a possible cause. However, since only a few cases were evaluated, the authors did not delve deeply into this topic [7]. The possibility of a reduction in nerve fiber layer thickness secondary to ischemic insult leading to decreased reflectance was also considered. Nonetheless, this was ruled out, as the hyporeflectant areas did not follow the anatomical distribution of the nerve fiber layer [7].

In the current study, we observed hyporeflectance in the retina on the BLR in non-perfused regions. Following ischemic damage caused by retinal non-perfusion, particularly in the DCP, the retina clinically reacts in the initial stages with grayish-white patches that return to normal coloration within a few weeks. On OCT, these areas appear hyper-reflective in the acute phase, progressing to reduced reflectivity and thinning (atrophy) of the inner retinal layers. When the deep capillary plexus is exclusively affected, the INL and OPL layers undergo atrophy in the late stages.

The findings from OCT and OCTA, correlated with the BLR findings in this study, suggest that hyporeflectant areas occur due to atrophy of the INL and OPL layers of the retina. This is supported by the following observations: (1) In all presented cases, structural OCT showed a correlation between hyporeflectant areas and the absence of INL and OPL. (2) Even in the case with normal perfusion of the SCP, as seen on OCTA, which displayed INL and OPL atrophy on structural OCT due to ischemia in the DCP, the hyporeflectant areas in the BLR correlated with the areas of INL and OPL atrophy.

These observations lead us to consider that the INL and OPL layers, either together or individually, play a fundamental role in the reflection of blue wavelength light. To the best of our knowledge, this is the first study that correlates BLR to specific retinal structures.

Given these findings, we believe that, just as the near-infrared (NIR) wavelength used in cSLO NIR reflectance imaging has a significant portion of its energy reflected by the interdigitation zone and consequently has the potential to facilitate the identification of diseases such as acute macular neuroretinopathy and hydroxychloroquine-induced drug toxicity, BLR has a specific interaction with the INL and OPL layers and has the capability to identify changes in these layers.

This unprecedented information is particularly noteworthy as it illustrates the potential for expanding the indications of the technique to conditions beyond diabetes and venous occlusions [5, 7, 8]. Our current study expands these preliminary observations by demonstrating the applicability of BLR imaging across a range of retinal non-perfusion scenarios.

Previous studies evaluating non-perfused areas using blue light reflectance were conducted with image acquisition via cSLO [5, 7], raising the question of whether these findings would be reproducible with other blue-light imaging methods [7]. The findings of the present study elucidate this issue, demonstrating the reproducibility of these results by decomposing the RGB channels from a CF image.

Unlike the cSLO system, which is expensive and not widely accessible, fundus photography is a widely used and cost-effective imaging technique. The use of digital filters to separate image channels is well-established and can be easily implemented without additional cost using embedded software in several retinal cameras or free software like ImageJ. For instance, in ImageJ, the command “Image -> Color -> Split Channels” allows for the separation of the BLR, enabling the identification of non-perfused areas [16].

BLR imaging may offer clinical utility in settings where access to OCT/OCTA is limited or for patients who cannot undergo contrast-enhanced imaging. The ability to identify non-perfused areas using readily available retinal cameras makes this technique particularly valuable in such contexts. Our findings suggest that BLR imaging can complement angiography by indicating areas of DCP ischemia, providing a more comprehensive evaluation of the retinal vascular meshwork without relying on OCT.

We highlight the critical role of ischemia in retinal diseases and underscore the positive impact of identifying such biomarkers. The potential of BLR imaging to detect DCP ischemia, even when SCP perfusion appears normal, presents a significant advancement in diagnosing retinal vascular diseases.

Furthermore, the observations of the present study explain the possible reason why a previous study noted a larger area of non-perfusion in fluorescein angiography compared to the BLR image [7]. Considering that hyporeflectance in the BLR occurs after the atrophy of the INL and OPL layers, and that this atrophy takes weeks to develop following the ischemic damage caused by retinal non-perfusion, it is plausible to speculate that recent hypoperfused areas seen in FA may not correspond to hyporeflective areas in the BLR. For instance, in Fig. 1, non-perfusion areas on FA appear larger than those seen in BLR images, which may be due to the time lag between ischemic insult and the development of retinal thinning detectable by BLR imaging. However, future research with longitudinal follow-up of these patients is necessary to confirm this hypothesis.

The findings of this study, particularly the correlation between hyporeflectant areas in BLR images and the absence of identifiable INL and OPL in structural OCT B-scans offer valuable insights into the complex interaction of blue light with retinal tissue. This process is dictated by the optical properties of blue light and the unique anatomical and biochemical characteristics of the inner retinal layers. Each layer possesses the inherent property of reflecting incident light, known as reflectivity, with blue light undergoing significant scattering due to its shorter wavelength and higher energy. This scattering is influenced by microstructural components, such as cylindrical organelles, and the thickness, density, and organization of the retinal architecture. The retina’s distinct structural and cellular layers generate specific reflectivity patterns, with high reflectivity seen in synaptic layers like the OPL, while nuclear layers such as the INL demonstrate lower reflectivity [17].

The OPL plays a crucial role in the retinal architecture and function by containing synapses among and between retinal photoreceptors, horizontal cells, and bipolar cells. Desmosome-like attachments called synaptic densities are located within the arrangement of interwoven [17]. These synaptic densities are seen as a series of dashed lines on light microscopy and resemble a discontinuous membrane, termed the middle limiting membrane, that might characterize resistance to the penetration of blue light. In retinal imaging, the presence of the OPL can influence the distribution and reflection of light, contributing to the overall reflectivity patterns seen in BLR.

Despite the strong theoretical basis suggesting the OPL as the most likely structure related to BLR, every analyzed image showed that the hyporeflectant areas consistently lacked both OPL and INL layers. Our study was unable to determine whether a single layer or a combination of both is primarily responsible for reflecting and scattering blue light.

While BLR imaging offers several advantages, it is important to acknowledge the broader limitations of this study. The sample size is relatively small, with only one or two cases representing each retinal disease, which restricts the generalizability of our findings and does not account for varying severities or stages of the diseases. Additionally, the correlation between different imaging modalities was not subjected to statistical analysis; instead, it was evaluated qualitatively. Furthermore, BLR imaging itself is subject to limitations, including interference from media opacities (e.g., cataracts), blue-light filtering intraocular lenses, and prior use of fluorescein eye drops, which can create image artifacts [18]. These factors can affect the clarity and accuracy of BLR images, necessitating careful consideration when interpreting results [12]. Discoloration in ischemic areas can be seen in color fundus images, and while challenging, some retina specialists can detect these changes. We emphasize that this study did not aim to evaluate the sensitivity nor specificity of the detection of these findings using BLR, but rather to show that the identification of non-perfusion is within a range of retinal non-perfusion scenarios.

In addition, BLR acquired from color fundus photography is valuable for detecting retinal nerve fiber layer defects in glaucomatous patients, often outperforming red-free imaging [19, 20]. However, it should be complemented by other modalities like OCT and FA to assess deeper retinal layers and differentiate between ischemic and non-ischemic retinal damage.

This study reinforces the evidence, through OCT, OCTA, and angiographic correlation, that the BLR can effectively identify areas of retinal non-perfusion in a non-invasive manner. Future research should explore the interaction of different light wavelengths with retinal tissue to enhance our understanding of retinal anatomy and physiology. This could lead to improved diagnostic capabilities and potentially uncover new therapeutic targets. In conclusion, BLR imaging presents a promising, cost-effective, and non-invasive method for detecting retinal non-perfusion, with significant implications for clinical practice, particularly in resource-limited settings and for patients unable to undergo contrast-enhanced imaging.

## Data Availability

No datasets were generated or analysed during the current study.
